# Longitudinal study of somatic development in Polish patients with Silver-Russell syndrome reveals that chest-to-head proportion as a new clinical indicator for the syndrome

**DOI:** 10.1186/s13023-026-04395-2

**Published:** 2026-05-20

**Authors:** Anna Świąder-Leśniak, Dorota Jurkiewicz, Agnieszka Madej-Pilarczyk, Krystyna Chrzanowska

**Affiliations:** 1 Laboratory of Anthropology, The Children’s Memorial Health Institute, Al. Dzieci Polskich 20, Warsaw, 04-730 Poland; 2 Department of Medical Genetics, The Children’s Memorial Health Institute, Warsaw, Poland

**Keywords:** Silver-Russell syndrome, Physical development, Chest-to-head proportion

## Abstract

**Background:**

Silver-Russell syndrome (SRS) is a rare congenital imprinting disorder associated with the loss of methylation in *H19/IGF2*:IG-DMR at chromosome 11p15.5 (11p15 LOM) or maternal uniparental disomy of chromosome 7 (upd(7)mat).

**Methods:**

Longitudinal analysis of somatic development was performed in 99 Polish patients with Silver-Russell syndrome: 82 (82.8%) with 11p15 LOM, 17 (17.2%) with upd(7)mat, without growth hormone therapy. All patients were assessed by a clinical geneticist and molecular tests were taken. Birth parameters were analyzed. Weight, height, BMI, and chest-to-head proportions were collected cross-sectionally and in different stages of development (every year, until the age of 16-18y).

**Results:**

ROC analysis showed good diagnostic performance of head–chest proportionality indices in distinguishing SRS patients from SGA patients at birth. The difference between head and chest circumference demonstrated an AUC of 0.840 (95% CI 0.768–0.912), increasing to 0.850 (95% CI 0.771–0.929) after exclusion of preterm infants, while the chest-to-head ratio showed an AUC of 0.812 (95% CI 0.732–0.892) and 0.827 (95% CI 0.741–0.912), respectively. These simple indices, derived from routine neonatal measurements, may support early identification of SRS among SGA infants. Moreover chest-to-head circumference proportion in SRS patients does not change until the age of 9 years in boys and 8 years in girls. Long-term studies showed that body height, weight, weight-for-height, and BMI were higher in patients with 11p15LOM up to the age of 10-12; however, this trend reverses later in favor of the upd(7)mat group.

**Conclusion:**

The introduction of head and chest circumference measurement into routine clinical examination may be useful, especially when the diagnosis of SRS is based solely on clinical symptoms. Long-term studies have shown a tendency for changes in body weight, height, and BMI depending on age and molecular abnormalities.

**Supplementary Information:**

The online version contains supplementary material available at 10.1186/s13023-026-04395-2.

## Background

Silver-Russell syndrome (SRS, OMIM #180860) is a rare imprinting disorder associated with the loss of methylation in *H19/IGF2*:IG-DMR at 11p15.5 (11p15 LOM), found in 35–67% of patients, or maternal uniparental disomy of chromosome 7 (upd(7)mat), found in 7–10% of patients [1–5]. The molecular etiology remains unknown in about 40% of patients with clinical symptoms of SRS [6]. The syndrome was first described by Silver et al. [7] and Russell [8].

Patients with SRS are characterized by intrauterine and postnatal growth retardation, without catch-up [4, 9–11]. A wide spectrum of minor dysmorphic features is observed in children with SRS, including a triangular face, a small mandible, crowded teeth, down-turned mouth, low-set and/or posteriorly rotated ears, clinodactyly of the fifth finger and/or syndactyly of 2–3 toes, delayed closure of the frontal fontanel, low muscle mass, excessive sweating or spinal deformity [4, 5, 10, 12–17]. The first International Consensus on the diagnosis and management of SRS recommends the Netchine-Harbison clinical scoring system (NH-CSS) [11].

Physical development in SRS patients has been investigated for many years. In 1975, Tanner [18] described the natural history of the disease. Twenty years later, Wollmann [9] published a mixed longitudinal and cross-sectional analysis of 386 SRS children. To treat short stature in SRS patients, recombinant human growth hormone (rhGH) was introduced in the last decade of the 20th century [19–21].

We present the first retrospective-prospective longitudinal study of 99 patients with genetically confirmed SRS, without growth hormone therapy, aiming to compare differences in physical development between patients with 11p15LOM and upd(7)mat.

## Study design

### Patients

The study group consisting of 99 patients (55 boys, 44 girls, aged 0-18y) was divided into two subgroups: 82 (82.8%) patients with 11p15 LOM and 17 (17.2%) with upd(7)mat. Birth data of SRS patients has been compared with SGA (small for gestational age) control group (29 patients: 14 boys and 15 girls). The study was conducted in accordance with the Declaration of Helsinki and approved by the institutional Bioethics Committee. Informed consent was obtained from all individual participants included in the study.

## Methods

The diagnostics and follow-up of patients with SRS at The Children’s Memorial Health Institute (CMHI) in Warsaw has been carried out since 1981. All patients were assessed by a clinical geneticist. Our patient cohort represents the largest group of patients with a diagnosis of SRS confirmed by molecular testing in Poland and one of the largest in Europe. Many years of research allowed us to describe in details the process of physical development and growth of SRS patients. To ensure homogeneity of the group in this study we included only anthropometric measurements of 99 SRS patients who had not been treated with growth hormone (GH). In our patient cohort, 44 individuals (25 boys and 19 girls) initiated growth hormone therapy; however, for the purposes of this publication, only anthropometric data collected prior to GH treatment were included.

Retrospective data from the patients’ medical records, i.e., body weight and length/height, head and chest circumference, were also analyzed. Selected parameters were collected in different stages of development (every year, until the age of 16-18y). Repeated longitudinal assessments were available for 92 patients, whereas 7 patients were assessed at a single time point.

The number of measurements for each parameter varied, i.e. weight measurement was recorded an average of 12 times per patient (median 10), length/height measurement an average of 10 times (median 9). The average age of initiation of rhGH treatment was 6.31 ± 2.45 years (median 6.04 years, range: 1.42–12.7 years), therefore research possibilities in the later stages of ontogenesis were limited because of decrease with age the amount of measurements for individual features. Analyzes for height, weight, weight for age and BMI were carried out cross-sectionally and in age categories every 1 year, excluding the last stage of ontogeny (the 17-years-old category included measurements performed between 16 and 18 years). Additionally, the category of 0.25 years (3 months) was created. Measurements of body proportions were analyzed cross-sectionally in relation to calendar age and age for height, defined as the age at which a patient’s body height corresponds to the 50th percentile.

Due to the risk of hypoglycemia in patients with Silver–Russell syndrome, a comparative analysis was performed between patients with reported episodes of hypoglycemia and those without such episodes. Birth and postnatal head–chest anthropometric proportions were analyzed. For postnatal data, a cross-sectional analysis was performed based on the first available anthropometric measurements of head and chest circumference.

### Birth parameters

Birth parameters were obtained from patients’ medical records and were standardized according to gender and gestational age [22]. Relative macrocephaly at birth was defined as a head circumference ≥ 1.5 SDS above birth length and/or weight [11]. SGA was defined as a birth body weight ≤-2 SDS [23], premature birth was defined as born before 37 weeks of amenorrhea. Based on head and chest circumference measurements, the chest-to-head ratio for SRS and SGA patients were calculated using formula: [head circumference (cm)/ chest circumference (cm)]×100.

### Measurements

**Fig. 1:**
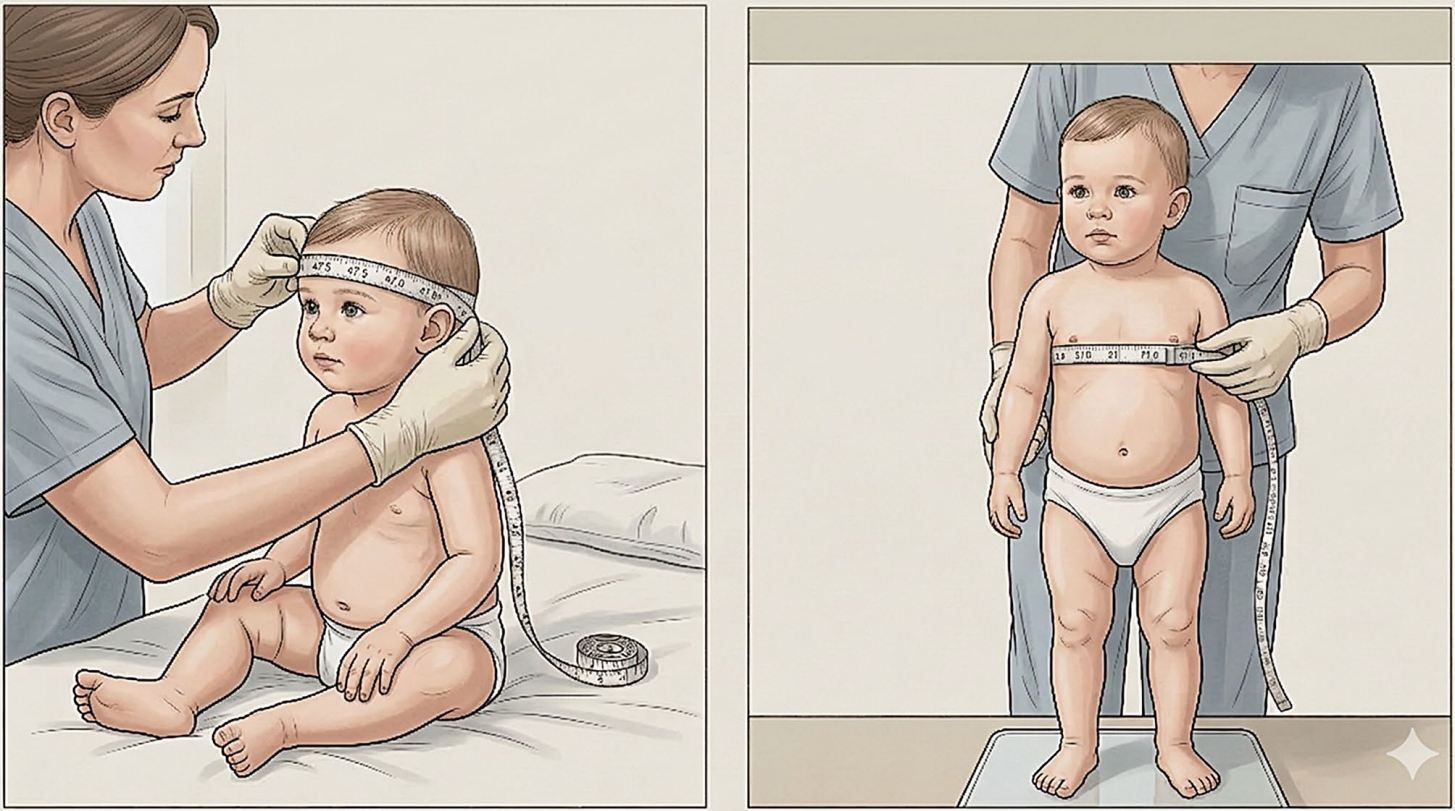
Anthropometric technique for measuring head and chest circumference

Anthropometric measurements were taken according to a standard technique [24]. Until the age of 2 years, the children’s body length was measured with a liberometer in the supine position; height in older patients was measured with a stadiometer in standing position; the result was the average of 3 independent measurements (compensated for the shorter limb in the case of body asymmetry). Body weight was measured using a medical scale accurate to 0.01 kg. A non-stretchable tape was used to assess chest and head circumference. Chest circumference was measured in the horizontal plane at the level of the *xiphoidale* point. Head circumference was measured at the level of the *methopion* and *opisthocranion* points. Body proportions were measured using standard equipment: GPM (Gneupel Präzisions-Mechanik) calipers, and an anthropometer. The following parameters were obtained: trunk length *sst-sy* (*suprasternale* to *symphysion*), lower extremity length *B-sy* (between *basis* and *symphysion*), upper extremity length *a-daIII* (between *acromion* and *dactylion III*), shoulders width *a-a* (biacromial diameter *acromion-acromion*), chest width thl-thl (bithoracolaterale diameter *thoracolaterale- thoracolaterale*), chest depth *xi-ths* (between *xiphoidale* and *thoracospinale*) and hip width *ic-ic* (*iliocristale-iliocristale*). All measurements were standardized for age and gender using Polish reference charts [25]. Figure 1 shows the measurement of head and chest circumference.

### Molecular analysis

Genomic DNA was extracted from peripheral blood leukocytes. Methylation-sensitive multiplex ligation-dependent probe amplification (MS-MLPA) using the SALSA MLPA KIT ME030BWS/SRS (MRC-Holland, Amsterdam, Netherlands) was carried out according to the manufacturer’s instructions. Data were analyzed using GeneMarker software, v.1.8 (Soft Genetics LLC, State College, PA, USA). Microsatellite typing was performed using polymorphic markers for chromosome 7: D7S507 (7p21), D7S460 (7p14), D7S663 (7q11), and D7S820 (7q21).

### Statistical analysis

Statistical analysis was carried out using Statistica 13.3 software. Data normality was analyzed using the Shapiro-Wilk test. The homogeneity of variance was checked using Levene’s test and the Brown-Forsythe test. Differences between the examined groups were analyzed using Student’s t-test or the Mann-Whitney U test. Values were expressed as mean and 95% confidence intervals for normal distribution and as median and quartile (Q1/Q3) for skewed distribution. The chi-square test was used to compare the number of patients in groups and the frequency of comparison characteristics. Receiver operating characteristic (ROC) analysis for birth parameters such as the difference between head and chest circumference (HC-CHC), the chest-to-head ratio (CHC-HC ratio), and macrocephaly calculated as the difference between head and weight SDS (RMC weight) and as the difference between head and length SDS (RMC length) was performed, in order to determine the diagnostic performance of these parameters and assess their usefulness in distinguishing between SRS and SGA patients. The area under the curve (AUC) with 95% confidence intervals was calculated. The Youden’s index was calculated to find the best cut-off points. ROC analysis were performed: (1) for all SRS and SGA patients, and (2) for group with premature newborns excluded. P-values < 0.05 were considered as statistically significant.

## Results

Table 1 shows the birth parameters of SRS patients and SGA control group. The number of premature babies was lower in the 11p15 LOM group than in the upd(7)mat group: 16 (19.5%) vs. 8 (47%), respectively, *p* = 0.04. The mean SDS of birth weight was significantly lower in patients with 11p15 LOM vs. upd(7)mat vs. SGA: − 4.29 vs. − 3.35 vs. -2.76 SDS, respectively, *p* = 0.015. Birth length (SDS) was the lowest in the 11p15 LOM group according to upd(7)mat and SGA: -2.37 vs. − 1.58 vs. -1.12 SDS, respectively (*p* = 0.014).

Head circumference SDS at birth did not differ significantly between the two SRS molecular subgroups, but was significantly lower in the SGA group in the overall comparison (*p* < 0.001). In contrast, chest circumference and the HC–CHC difference at birth were comparable between 11p15LOM and upd(7)mat patients, while both parameters differed significantly when compared with the SGA group (*p* < 0.001). Similarly, the CHC-HC ratio was significantly higher in SRS patients than in the SGA group (*p* < 0.001), with no significant difference between the two SRS molecular subgroups.

**Table 1:** Birth parameters of patients with Silver-Russell syndrome

Parameter	SRS *n* = 99 [95% CI]/ (Q1-Q3)	11p15LOM *n* = 82 [95% CI]/ (Q1-Q3)	upd(7)mat *n* = 17 [95% CI]/ (Q1-Q3)	SGA *n* = 29 [95% CI]/ (Q1-Q3)	*p* 11p15LOM vs. upd(7)mat	*p* 11p15LOM vs. upd(7)mat vs. SGA
Premature (%)	24 (24.3)	16 (19.5)	8 (47.0)	5 (17.2)	**0.04**	**0.04**
Pregnancy week	37.9 [37.5–38.4]	38.3 [37.8–38.7]	36.4 [35.0–37.9]	37.6 [36.5–38.8]	**0.002**	**0.015**
Birth weight (SDS)	-4.13 [-4.44 – (-3.82)]	-4.29 [-4.61 – (-3.97)]	-3.35 [-4.26 – (-2.44)]	-2.76 [-2.99 – (-2.52)]	**0.023**	**0.047**
Birth length (SDS)	-2.24 [2.57 – (-1.91)]	-2.37 [-2.74 – (-2.00)]	-1.58 [-2.28 – (-0.88)]	-1.12 [-1.5 – (-0.74)]	0.08	**0.014**
Birth HC (SDS)	-1.13 [-1.35 – (-0.91)]	-1.04 [-1.28 – (-0.79)]	-1.55 [-2.06 – (-1.04)]	-2.33 [-2.8 – (-1.9)]	0.08	**< 0.001**
Birth CHC (cm)	26.9 [26.2–27.5]	27.1 [26.3–27.8]	25.9 [24.4–27.3]	28.0 [27.0–29.1]	0.16	0.08
HC-CHC* (cm) at birth	5.0 (4.0–7.0)	5.0 (4.0–8.0)	4.5 (4.0-5.5)	2.0 (1.0–4.0)	0.91	**< 0.001**
CHC-HC ratio (%)	121.9 [119.4–124.5]	122.3 [119.4–125.3]	120.0 [116.0–124.1]	108.5 [105.7–111.2]	0.35	**< 0.001**

*The difference between head and chest circumference in patients born ≥ 37 weeks of amenorrhea

11p15LOM - loss of methylation *H19/IGF2*:IG-DMR at chromosome 11p15.5;95% CI -95% confidence interval, CHC - chest circumference, CHC-HC ratio - chest-to-head circumference ratio

HC - head circumference, HC-CHC – difference between head and chest circumference, Q1 - first quartile, Q3 - third quartile, SDS - standard deviation score, SGA - small for gestational age, SRS - Silver-Russell syndrome,

upd(7)mat - uniparental disomy of chromosome 7

Fifty-seven patients with 11p15 LOM (69.5%) and 10 patients with upd(7)mat (58.8%) met at least 4 of the NH-CSS criteria, including relative macrocephaly and protruding forehead (*p* = 0.028) (Table 2). Body asymmetry was identified more often in the group with 11p15 LOM vs. the upd(7)mat group: 86.1% vs. 25% (*p* < 0.001). There were no statistically significant differences between the groups analyzed for the following traits: SGA, postnatal growth failure, relative macrocephaly, protruding forehead, feeding difficulties, and/or low BMI (Table 2).

**Table 2:** Netchine-Harbison clinical scoring system and frequency of clinical features in patients with Silver-Russell syndrome

Clinical criteria	SRS (%)	11p15 LOM (%)	upd(7)mat (%)	*p*
**Netchine-Harbison clinical scoring system**				
(1) SGA	93/99 (93.9)	78/82 (95.1)	15/17 (88.2)	0.60
(2) Postnatal growth failure	95/97 (97.9)	78/80 (97.5)	17/17 (100)	0.78
(3) RMC at birth	78/ 97 (80.4)	66 / 80 (82.5)	12/17 (70.6)	0.43
(4) Protruding forehead	86/96 (89.6)	72/80 (90.0)	14/16 (87.5)	0.88
(5) Body asymmetry	72/95 (75.8)	68/79 (86.1)	4/16 (25.0)	**< 0.0001**
(6) Feeding difficulties and/or low BMI	74/96 (77.1)	60/79 (75.9)	14/17 (82.3)	0.80
4 NH-CSS including (3,4)	67/97 (69.0)	57 (69.5)	10 (58.8)	**0.03**
**Additional clinical features of SRS**				
Face asymmerty	51/80 (63.7)	46/64 (71.9)	5/16 (31.3)	**0.003**
HC postnatal 3–97 pc.	78/98 (79.6)	63/82 (76.8)	15/16 (93.8)	0.23
Microcephaly*	12/97 (12.4)	10/80 (12.5)	2/17 (11.8)	0.75
Triangular face	85/90 (94.4)	71/73 (97.3)	14/17 (82.4)	0.07
Micrognathia	84/92 (91.3)	69/75 (92.0)	15/17 (88.2)	0.98
Down-turned mouth	77/87 (88.5)	64/71 (90.1)	13/16 (81.3)	0.57
Low set and/or posteriorly rotated ears	41/69 (59.4)	33/58 (56.9)	8/11 (72.7)	0.52
Fifth finger clinodactyly	53/85 (74.1)	53/70 (75.7)	10/15 (66.6)	0.69
Syndactyly of toes	35/60 (58.3)	32/48 (66.6)	3/12 (25.0)	**0.009**
Hypoglycaemia	19/67 (28.4)	19/55 (34.5)	0/12 (0)	**0.04**
Excessive sweating	61/82 (74.4)	48/67 (71.6)	13/15 (86.6)	0.38
Speech dealy	22/38 (57.9)	15/29 (51.7)	7/9 (77.7)	0.32
Motor dealy	38/75 (50.7)	32/61 (52.5)	6/14 (42.9)	0.52

Microcephaly* - defined as a head circumference 2 or more SDs less than the mean for age and sex,

11p15LOM - loss of methylation *H19/IGF2*:IG-DMR at chromosome 11p15.5, BMI – body mass index, HC - head circumference, NH-CSS - Netchine-Harbison clinical scoring system, pc – percentile, RMC – relative macrocephaly, SDS - standard deviation score, SGA - small for gestational age, SRS - Silver-Russell syndrome

upd(7)mat - uniparental disomy of chromosome 7

Several other clinical features were recognized in SRS patients (Table 2) that are not specific to SRS alone but might be helpful in clinical diagnosis. Face asymmetry, syndactyly of toes, and hypoglycemia were identified significantly more often in the 11p15LOM group than in the upd(7)mat group: 71.9% vs. 31.3% (*p* = 0.003), 66.6% vs. 25.0% (*p* = 0.009) and 34.5% vs. 0.00% (*p* = 0.04), respectively.

Birth and postnatal anthropometric parameters were compared between patients with and without hypoglycemia. At birth, no significant differences were observed between the two groups in head circumference SDS, HC–CHC difference, or the chest-to-head ratio. Analysis of the first available postnatal measurements also showed no significant differences between patients with and without hypoglycemia (Table 3).

**Table 3:** Birth and postnatal head–chest anthropometric proportions in patients with Silver–Russell syndrome with and without hypoglycemia

Parameter	SRS hypoglycemia Mean [95% CI]	SRS No hypoglycemia Mean [95% CI]	*p* (t-test)
**Birth data**			
HC (SDS)	−0.82 [− 1.35 – (− 0.29)]	−0.68 [− 0.97 – (− 0.39)]	0.619
HC–CHC difference (cm)	5.31 [3.83–6.79]	5.68 [4.86–6.50]	0.650
CHC-HC ratio (%)	121.27 [114.30–128.24]	122.63 [118.85–126.41]	0.722
**Postnatal data**			
HC (SDS)	−1.06 [− 1.69 – (− 0.43)]	−1.12 [− 1.44 – (− 0.80)]	0.855
CHC (SDS)	−3.71 [− 4.45 – (− 2.97)]	−3.55 [− 3.95 – (− 3.15)]	0.696
HC–CHC difference (cm)	5.44 [4.76–6.13]	5.68 [4.95–6.42]	0.629
CHC-HC ratio (%)	115.41 [112.88–117.94]	115.22 [113.26–117.18]	0.902

95% CI – 95% confidence interval, CHC – chest circumference, HC – head circumference

HC–CHC – difference between head and chest circumference (cm), CHC/HC – chest-to-head circumference ratio, p – p- value, SRS – Silver-Russell syndrome

**Fig. 2:**
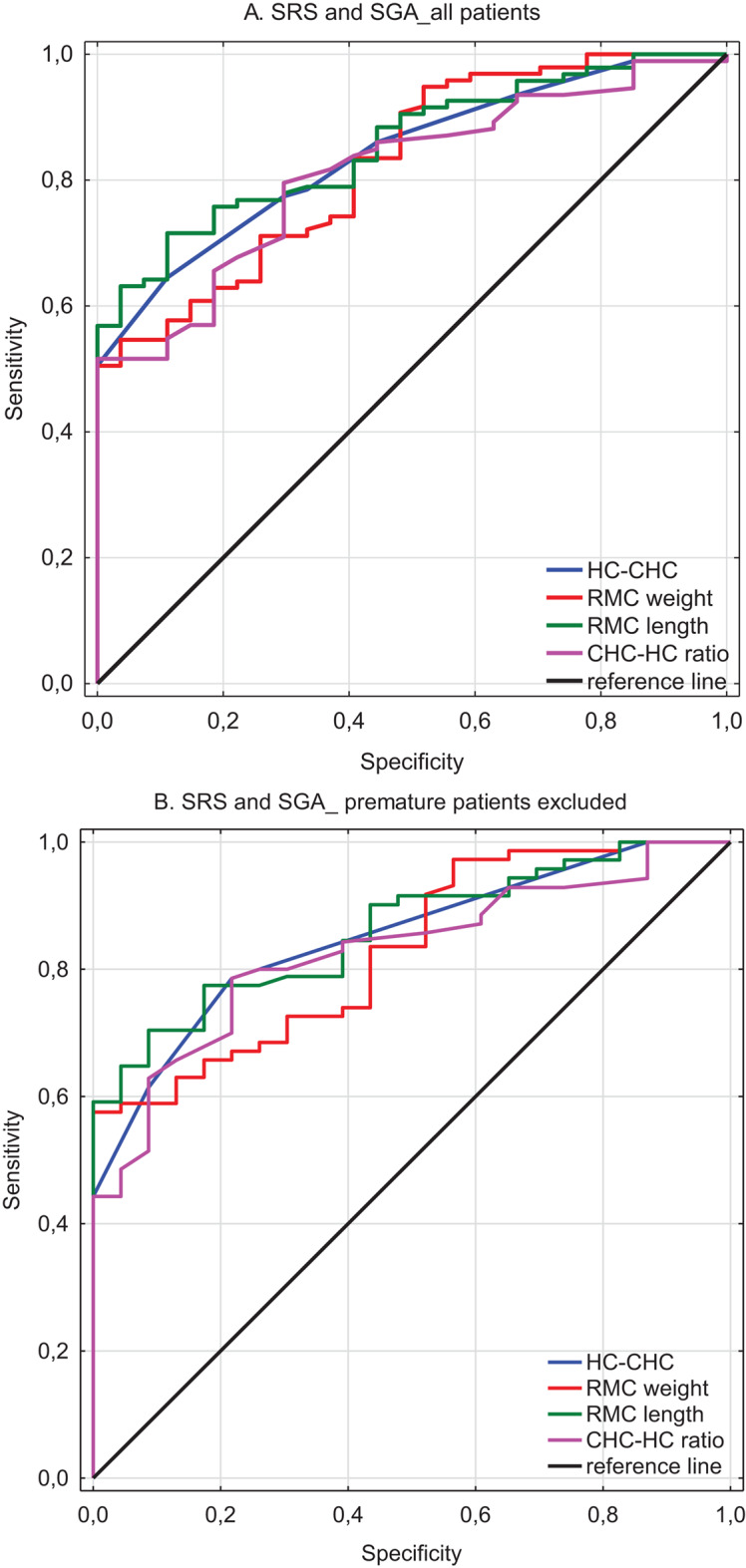
Receiver operating characteristic curves (ROC) for birth parameters in patients with SRS and SGA: (**A)** all patients; (**B**) premature patients excluded. Abbreviations: CHC-HC ratio - chest-to-head ratio, HC-CHC– difference between head and chest circumference, RMC – relative macrocephaly, ROC- receiver operating characteristic, SRS – Silver-Russell syndrome, SGA – small for gestational age

Receiver operating characteristic (ROC) analysis was conducted to assess the diagnostic performance of birth anthropometric parameters for Silver–Russell syndrome in the entire cohort and after exclusion of preterm infants (Table 4; Fig. 2). In the entire cohort, all analyzed anthropometric parameters demonstrated good discriminatory performance in ROC analysis, with AUC values ranging from 0.812 to 0.855 (*p* < 0.001). The highest diagnostic accuracy was observed for RMC length (AUC 0.855, 95% CI 0.788–0.922), followed by HC–CHC difference (AUC 0.840, 95% CI 0.768–0.912), RMC weight (AUC 0.826, 95% CI 0.747–0.905), and CHC–HC ratio (AUC 0.812, 95% CI 0.732–0.892). RMC length showed the highest Youden index (0.605), indicating the most favorable balance between sensitivity and specificity. After exclusion of preterm neonates, the discriminatory performance of all parameters remained stable, with AUC values ranging from 0.827 to 0.861 (*p* < 0.001). RMC length continued to demonstrate the highest accuracy (AUC 0.861, 95% CI 0.788–0.935; Youden index 0.617), maintaining a balanced sensitivity (0.704) and specificity (0.913). Importantly, HC–CHC difference (AUC 0.850) and CHC–HC ratio (AUC 0.827) also retained good and clinically meaningful discriminatory capacity. The exclusion of preterm neonates resulted in only minor changes in the optimal cut-off values for HC–CHC (5.0 to 4.0), CHC–HC ratio (120 to 112.9), and RMC weight (1.818 to 2.072), while the cut-off for RMC length remained unchanged (− 0.009), with overall diagnostic performance remaining comparable between cohorts. Overall, exclusion of preterm neonates did not materially alter ROC characteristics, indicating that gestational age did not significantly influence the observed diagnostic performance.

**Table 4:** ROC analysis of the diagnostic performance of anthropometric parameters for Silver-Russell syndrome in the entire cohort and after exclusion of premature infants

Parameter	Cohort	AUC [95% CI]	*p*-value	Cut-off	Sensitivity	Specificity	Youden index
HC–CHC	1	0.840 [0.768–0.912]	< 0.001	5.0	0.645	0.889	0.534
	2	0.850 [0.771–0.929]	< 0.001	4.0	0.786	0.783	0.568
CHC–HC ratio	1	0.812 [0.732–0.892]	< 0.001	120.0	0.516	1.000	0.516
	2	0.827 [0.741–0.912]	< 0.001	112.9	0.786	0.783	0.568
RMC weight	1	0.826 [0.747–0.905]	< 0.001	1.818	0.546	0.963	0.509
	2	0.829 [0.744–0.914]	< 0.001	2.072	0.575	1.000	0.575
RMC length	1	0.855 [0.788–0.922]	< 0.001	−0.009	0.716	0.889	0.605
	2	0.861 [0.788–0.935]	< 0.001	−0.009	0.704	0.913	0.617

1 – entire cohort, 2- excluding preterm

95% CI – 95% confidence interval, AUC – area under the receiver operating characteristic curve, CHC-HC ratio - chest-to-head circumference ratio, HC-CHC – difference between head and chest circumference (cm), RMC – relative macrocephaly, ROC - receiver operating characteristic

**Fig. 3:**
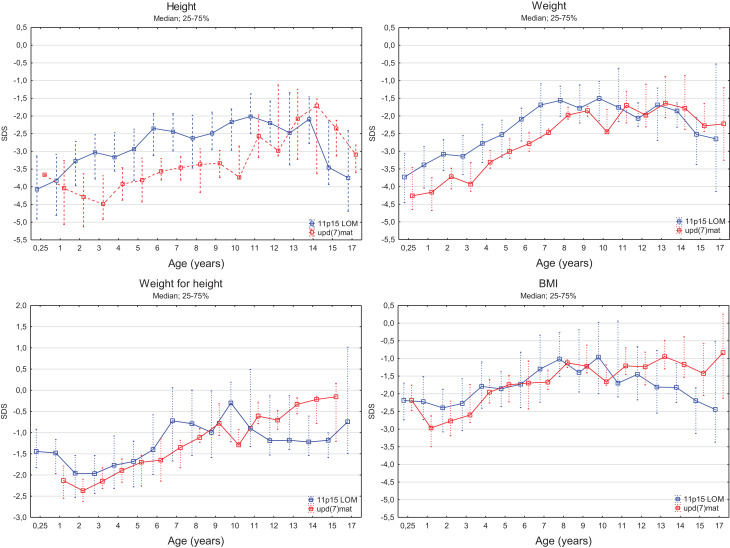
Height, weight, weight-for-height, BMI: a comparison between 11p15LOM and upd(7)mat patients in particular age categories. Abbreviations: 11p15 LOM – loss of methylation *H19/IGF2*:IG-DMR at chromosome 11p15.5, BMI – body mass index, upd(7)mat – uniparental disomy of chromosome 7

Height, weight, weight-for-height, and BMI were assessed cross-sectionally and by age categories. The median of age was 4.32 years for patients with 11p15LOM and 4.47 years for upd(7)mat patients. Patients with 11p15LOM had significantly higher values of height (-3.03 vs. -3.67 SDS; *p* < 0.001), weight (-2.79 vs. -3.13 SDS; *p* < 0.001), and weight-for-height (-1.48 vs. -1.67 SDS; *p* = 0.03) (Table 5). Long-term studies showed that body height was higher in patients with 11p15LOM between the ages 1 and 12 (significant difference from the 2 to 10 years-old group; Supplementary Table 1). Later, the trend changed, and from age 13 years onward body height was higher in the upd(7)mat group (Fig. 3). A similar trend was found for body weight, weight-for-height, and BMI (Fig. 3 and Supplementary Table 1).

**Table 5:** Comparison of anthropometric parameters in SRS patients depending on the molecular abnormality

	*N* of measurements 11p15LOM/upd(7)mat	11p15LOM SDS (Q1/Q3)	upd(7)mat SDS (Q1/Q3)	*p*
Age (years)	876/201	4.32 (1.80/7.58)	4.47 (2.58/8.60)	0.103
Height SDS	876/201	-3.03 (-3.88/-2.20)	-3.67 (-4.35/-3.03)	**< 0.001**
Weight SDS	1023/223	-2.79 (-3.50/-1.97)	-3.13 (-3.89/-2.37)	**< 0.001**
Weight for height SDS	849/198	-1.48 (-2.08/-0.81)	-1.67 (-2.20/-0.96)	**0.03**
BMI SDS	850/200	-1.95 (-2.57/-1.11)	-1.79 (-2.56/-1.29)	0.86
Head circumference SDS	601/131	-0.95 (-1.76/-0.22)	-0.26 (-1.45/0.65)	**< 0.001**
Chest circumference SDS	551/126	-2.73 (-3.80/-1.94)	-3.02 (-3.71/-2.15)	0.29
Chest-to-head ratio SDS	542/125	2.93 (1.93/4.12)	3.49 (2.67/4.31)	**0.002**

11p15LOM - loss of methylation *H19/IGF2*:IG-DMR at chromosome 11p15.5, BMI – body mass index, Q1 - first quartile, Q3 - third quartile, SDS - standard deviation score, SGA - small for gestational age, SRS - Silver-Russell syndrome, upd(7)mat - uniparental disomy of chromosome 7

**Fig. 4:**
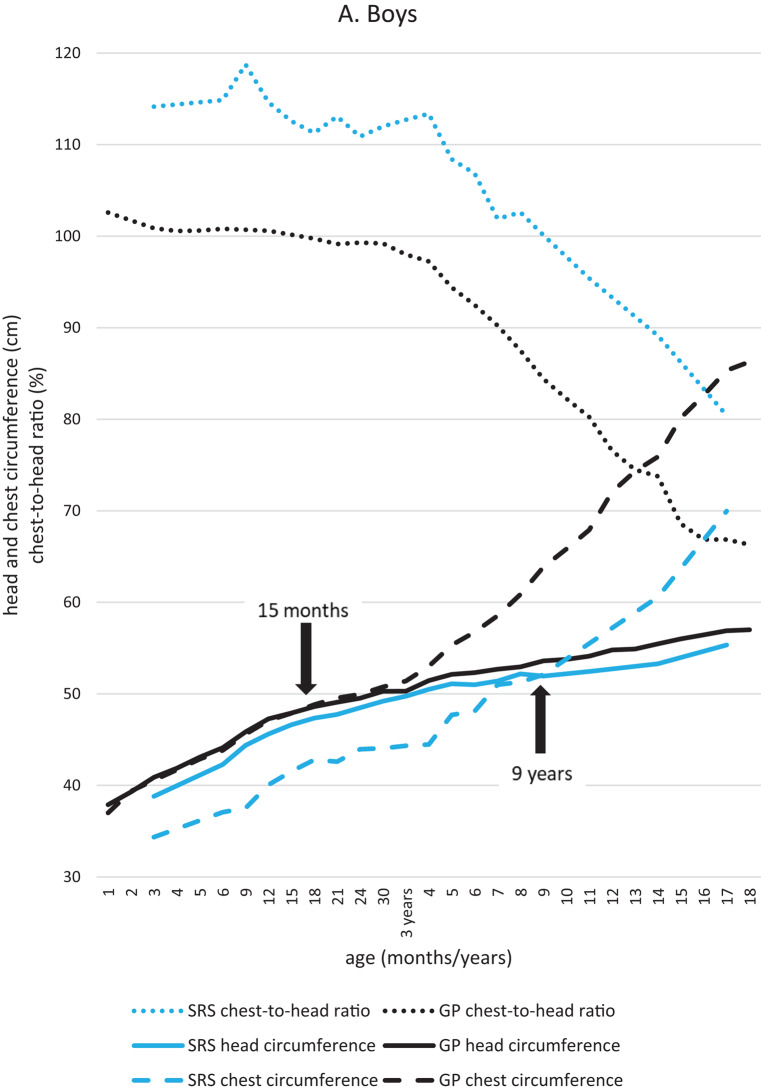

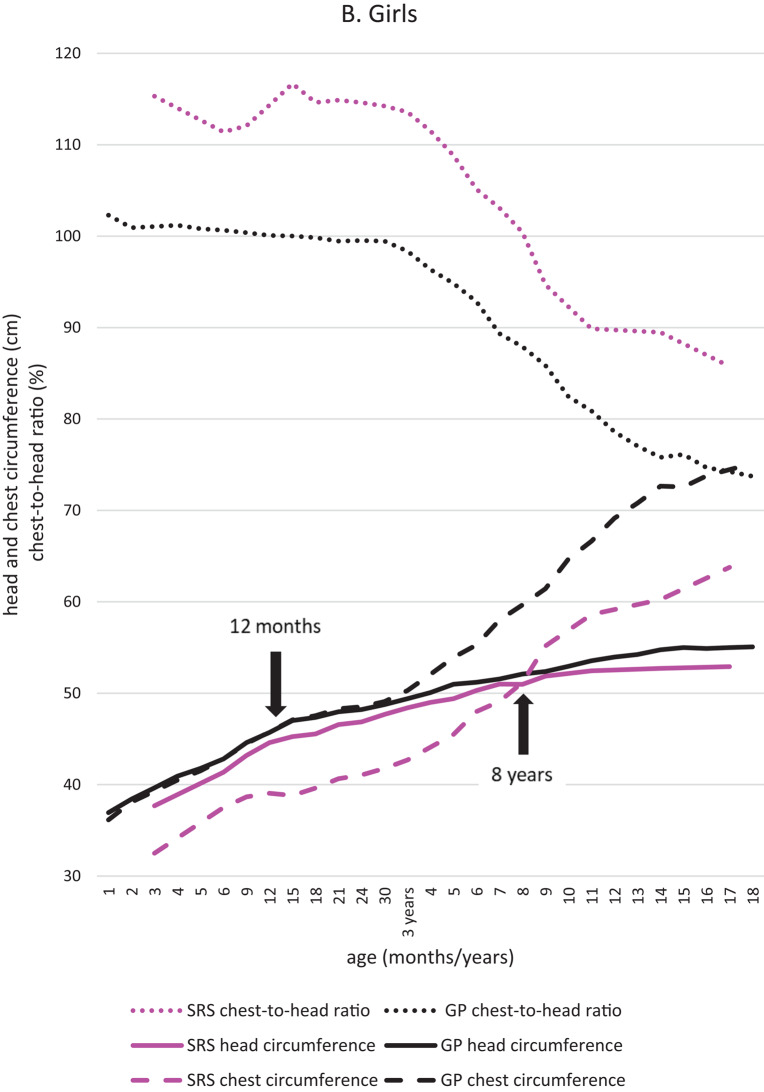
Difference between head and chest circumference in healthy children and SRS patients: A-boys; B-girls. Abbreviations: GP – general population, SRS – Silver-Russell syndrome

Cross-sectional analyses showed that patients with 11p15LOM had significantly smaller head circumference than patients with upd(7)mat (-0.95 vs. -0.26 SDS; *p* < 0.001) (Table 5). Chest circumference was similar in both groups (-2.73 vs. -3.02 SDS; ns). The chest-to-head ratio was significantly lower in patients with 11p15LOM (2.93 vs. 3.49 SDS; *p* = 0.002). This index was above the normal range in both analyzed groups. In healthy Polish children, the head circumference is bigger than the chest circumference up to the age of 15 months in boys and 12 months in girls [25]; then the chest circumference steadily increases in relation to the head circumference. We showed that the head circumference was bigger than the chest circumference in boys and girls with SRS until the age of 9 and 8 years, respectively (Fig. 4). The mean of the difference between head and chest circumference in the study group was 4.36 cm in boys and 4.82 cm in girls. In all age categories, the head circumference of boys and girls with SRS was significantly smaller than the head circumference of healthy peers (*p* < 0.05; mean difference in boys: 1.37 ± 0.42 cm; median: 1.32 cm; mean difference in girls: 1.33 ± 0.46 cm; median: 1.35 cm). The chest circumference of boys and girls with SRS was also significantly smaller in comparison to healthy children at each stage of physical development (*p* < 0.0001; mean difference in boys: 8.63 ± 2.96 cm; median: 7.53 cm; mean difference in girls: 7.86 ± 1.62 cm; median: 7.65 cm).

**Fig. 5:**
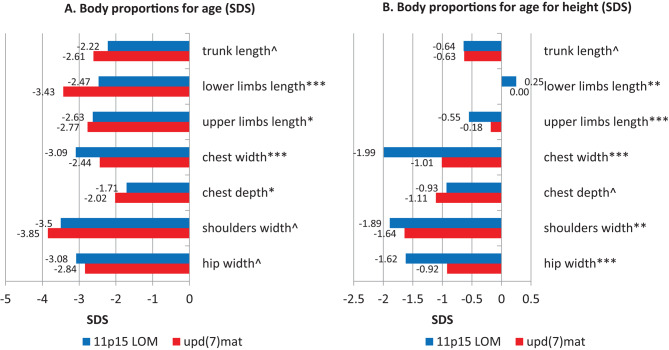
Body proportions for age (**A**) and age-for-height (**B**): a comparison between 11p15LOM and upd (7) mat patients. Abbreviations: 11p15 LOM – loss of methylation *H19/IGF2*:IG-DMR at chromosome 11p15.5, upd(7)mat – uniparental disomy of chromosome 7. ^ not statistically significant, * *p* < 0,05; ** *p* < 0,01; *** *p* < 0,001

Due to the short stature of SRS children, the body proportions were calculated for calendar age and for age-for-height (Fig. 5). Median measurements of the lower limbs, upper limbs, and trunk length, as well as body width, were below the population range (≤-2 SDS) in both 11p15 LOM and upd(7)mat groups (except for the chest depth measurement in the 11p15 LOM patients). The following were statistically significantly higher in 11p15LOM patients: lower limb length (-2.47 vs. -3.43 SDS; *p* < 0.001), upper limb length (-2.63 vs. -2.77 SDS; *p* < 0.05), and chest depth (-1.71 vs. -2.02 SDS; *p* < 0.05). Chest width was lower in the 11p15LOM group (-3.09 vs. -2.44 SDS; *p* < 0.001). With respect to age-for-height, body proportions such as trunk length, lower limb length, and upper limb length (SDS) were within the so-called normal narrow range (± 0.64 SDS, which corresponds to the range of 25–75 percentile) in both subgroups. Analysis of the normalized values ​​(SDS) of individual traits showed that 11p15 LOM patients had significantly: longer lower limbs (0.25 vs. 0.00 SDS; *p* = 0.001), shorter upper limbs (-0.55 vs. -0.18 SDS; *p* < 0.001), smaller chest width (-1.99 vs. -1.01 SDS; *p* < 0.001), smaller shoulder width (-1.89 vs. -1.64 SDS; *p* = 0.003), and smaller hip width (-1.62 vs. -0.92 SDS; *p* < 0.001) than patients with upd(7)mat.

## Discussion

The phenotype of patients with SRS is quite well characterized. Long-term follow-up at our center of a large group of SRS patients not treated with hrGH has made it possible to track physical development and assess differences according to the type of molecular abnormality (11p15 LOM vs. upd(7)mat). As in other studies [15, 21, 26–28], we showed that patients with 11p15 LOM had significantly lower birth body weight than patients with upd(7)mat. Head circumference at birth, which – unlike birth weight and length – is most often within the range of the population norm, was non-significantly higher in the 11p15 LOM group compared to the upd(7)mat group. The same relationship was indicated by other researchers [15, 27, 28]. It appears that measuring chest circumference at birth is routinely performed and documented only in Poland. To the best of our knowledge, data on chest circumference at birth in patients with SRS have not been reported in the world literature. In this study, we have shown that this parameter can help screen patients suspected of SRS. Considering only SRS patients born at term, the difference between head and chest circumference was 5 cm in the 11p15LOM group and 4.5 in the upd(7)mat group, respectively. In healthy children, the difference is about 1 cm^25^.

The Netchine-Harbison scale makes it possible to diagnose SRS when patients meet at least 4 out of 6 criteria, including the two mandatory ones, i.e., relative macrocephaly and protruding/prominent forehead. We showed that patients with 11p15 LOM were more likely to score ≥ 4 on the NH-CSS scale than patients with upd(7)mat (69.5% with 11p15 LOM vs. 58.8% with upd(7)mat; *p* = 0.028). These observations are in line with reports by other authors [5, 10, 12, 29]. The study group also included 22 patients who had 4–5 SRS features but with no protruding forehead and/or macrocephaly, and 10 patients meeting only 2–3 criteria according to the NH-CSS. In two patients who scored only 2 on the NH-CSS scale, the diagnosis was suspected based on the presence of a triangular facial shape or clinodactyly of the V fingers, which warranted referral for molecular testing that ultimately confirmed the diagnosis of SRS. Therefore, rigorous implementation of the NH-CSS criteria may result in failure to diagnose SRS or delayed diagnosis.

Hypoglycemia is one of the complications in patients with SRS and has been attributed to several mechanisms, including limited glycogen stores and increased metabolic demands. It has also been suggested that hypoglycemia may be related to a disproportion between brain size and hepatic glycogen reserves, with relatively larger brain size resulting in increased glucose consumption [11]. In this context, anthropometric parameters reflecting the relationship between head size and body size, such as the difference between head and chest circumference or the chest-to-head ratio, may theoretically serve as indicators of this disproportion. In our cohort we did not observe significant differences in these parameters between patients with and without hypoglycemia, either at birth or at the first postnatal measurement. These findings suggest that, in our cohort, anthropometric disproportion between head and chest circumference does not appear to be a major determinant of hypoglycemia risk. A limitation of this study is that information regarding hypoglycemia was obtained from questionnaires in which the occurrence of hypoglycemic episodes was recorded as present or absent. Quantitative measurements of blood glucose levels were not available. The lack of numerical glucose measurements limits the ability to assess the severity or frequency of hypoglycemia and therefore restricts the strength of conclusions regarding the relationship between anthropometric proportions and hypoglycemia.

The cross-sectional analyses showed that body height (median) was significantly lower in upd(7)mat vs. 11p15 LOM patients. Our results are consistent with research of Fuke [15] and Marsaud [30], who also showed that the body height of upd(7)mat patients were lower compared to 11p15 LOM patients (− 3.77 vs. − 3.58 SDS; mean age 4.8 and 4.1 years and − 3.6 vs. − 3.2 SDS, mean age 1.8 and 2.8, respectively). In contrast, Binder [26] showed that patients with 11p15 LOM had a lower body height vs. upd(7)mat patients. The study by Lokulo-Sodipe [28], which was conducted on an older group, also showed that patients with 11p15 LOM had a lower body height than patients with upd(7)mat. The discrepancies between the results of different authors are probably related to group size and median patient age. Despite the decreasing number of measurements in the older age classes, the long-term study provided important information on the changes in body height of patients with 11p15 LOM and upd(7)mat over time. It showed that body height (SDS) was lower in upd(7)mat patients in the younger age groups (1–12 years). In the older age groups (13–17 years), body height was lower in patients with 11p15 LOM. These findings emphasize the importance of longitudinal observations across the progressive period of ontogeny, which may explain discrepancies between previously published studies that were often based on smaller samples or cohorts of different ages.

Our cross-sectional analysis showed that the body weight was higher in the 11p15 LOM group than in the upd(7)mat group, both in terms of calendar age and in the weight-to-height relation. Other cross-sectional studies have shown that patients with 11p15 LOM had lower body weight vs. upd(7)mat patients [15, 26, 28, 30]. In our study, patients with upd(7)mat accounted for 47% of children born prematurely, which may have influenced their lower SDS values of body weight, especially at the beginning of follow-up. Long-term analyses by age classes (in terms of weight-for-age and weight-for-height) showed a similar trend to that for body height, i.e., in younger age classes, body weight (SDS) was higher in patients with 11p15 LOM, and in older age classes (from age 11), in patients with upd(7)mat. This trend was more pronounced in analyses relating to weight-for-height.

Cross-sectional analysis of BMI indicated no statistically significant differences between patients with 11p15 LOM and upd(7)mat. Long-term analysis across age categories showed that BMI (SDS) values in both upd(7)mat and 11p15 LOM patients increased steadily until the age of 11 years. At later stages of development, the SDS values remained relatively constant in the upd(7)mat group, but decreased with age in the 11p15 LOM group. In the upd(7)mat group, a relative increase in BMI was observed between the ages of 7 and 8, while in the 11p15 LO group, this increase occurred later, between the ages of 9 and 10. These differences may reflect the slightly earlier onset of puberty in patients with upd(7)mat. This observation is consistent with the findings reported by Patti [31].

While head circumference is usually measured at birth, such measurements are less frequent in the postnatal period. We showed that the median of head circumference (SDS) of SRS patients was within the population norm, regardless of the type of molecular abnormality, throughout the ontogenesis period analyzed. Cross-sectional analyses showed that head circumference was smaller in the 11p15 LOM group vs. the upd(7)mat group. Our results are consistent with those reported by other authors [15, 26].

We showed no statistically significant differences in chest circumference (SDS) between the 11p15LOM and upd(7)mat groups. Moreover, chest circumference (SDS) was below the range of reference values for a healthy population in both analyzed groups. The median values of head and chest circumference (SDS) indicated a disparity between these parameters: the head circumference of both the 11p15 LOM and upd(7)mat patients was within ± 1 SDS, while the chest circumference was below the population norm (below − 2.5 SDS). Considering these disparities and the differences between head and chest circumference, the chest/head ratio was calculated as the simplest scale describing the percentage ratio of head circumference in relation to chest circumference. The SDS chest/head ratio was significantly higher in the group with upd(7)mat (*p* = 0.002). Analyses of the correlation of head and chest circumference of SRS patients compared to the healthy population [25] showed that chest circumference was smaller than head circumference by the age of 9 years in boys and by the age of 8 years in girls with SRS. In healthy children, the chest circumference becomes greater than the head circumference much earlier – as early as 12–15 months of age. No data on chest circumference and chest/head ratio of patients with SRS were found in the literature (except in case reports), so the results presented in our study are unique. Given that patients with SRS are most often diagnosed in the first years of life when differences between head and chest circumference are the greatest, it is worth considering including these two measurements in the routine clinical examination.

The diagnosis of Silver-Russell syndrome is currently based on clinical features assessed using the Netchine-Harbison clinical scoring system (NH-CSS). However, some features included in this scale can only be assessed after the age of 24 months. Therefore, identifying clinical features that may suggest or support the diagnosis of SRS earlier in life is important for clinicians [32]. In our study, we indicated that the assessment of the head-to-chest proportions may be useful in the diagnosis of SRS.

In this study, birth anthropometric parameters based on head and chest measurements demonstrated good discriminatory ability in differentiating patients with SRS from SGA controls. All analyzed indices showed statistically significant diagnostic performance, with AUC values exceeding 0.80, indicating good overall accuracy. Importantly, proportional indices based on head and chest measurements, including HC–CHC difference and CHC–HC ratio, also demonstrated good and clinically meaningful discriminatory capacity. These parameters may reflect the characteristic relative macrocephaly observed in SRS, where head circumference is relatively preserved compared with other body measurements. These findings are consistent with the known clinical phenotype of SRS, in which relative macrocephaly at birth represents one of the key diagnostic features included in the Netchine–Harbison clinical scoring system.

Previous studies have shown that infants with SRS often present with a relatively preserved head circumference compared with reduced body weight and length, reflecting disproportionate growth restriction. Consequently, anthropometric indices that capture proportional differences between head circumference and other body measurements may be particularly useful for identifying this characteristic growth pattern [11, 16]. Our results support this concept, demonstrating that parameters reflecting head–chest proportions i.e. HC-CHC difference and chest-to-head ratio, can effectively distinguish SRS patients from SGA infants, a group that often presents with similar overall growth restriction but without the characteristic disproportionality observed in SRS. Since these indices are simple to obtain and require only routine neonatal measurements, they may serve as practical screening markers in the early assessment of neonates suspected SRS.

The body proportions of SRS patients were first presented by Russell [8] who noticed shortened upper limbs and relatively long lower limbs, as well as a narrow chest; this was based on a group of only five patients. The length of the lower and upper limbs was also assessed by Tanner [18], but this analysis was mainly used to assess body asymmetry. We showed that trunk length, and lower and upper limb length in relation to body height, were within the population range (25–75 pc, corresponding to ± 0.67 SDS), regardless of the type of molecular abnormality. This means that patients with SRS were characterized by proportional growth of the limbs and trunk in relation to body height. In contrast, the width of the chest, shoulder, and hips ranged from − 1.62 to -1.99 SDS in the 11p15 LOM group and from − 0.92 to -1.64 SDS in the upd(7)mat group, compared to the normal range. We also pointed out several differences in body proportions in SRS patients, regardless of molecular abnormality (Fig. 3).

In the study, we presented results for SRS patients not treated with growth hormone. On the one hand, this is a limitation due to the decreasing number of patients in the oldest age groups, but on the other hand, this approach allowed us to obtain unique results.

## Conclusions

We presented the first cross-sectional and long-term study that provides the physical development of SRS patients without growth hormone therapy. In longitudinal studies, we showed differences in height, weight, weight-for-height, and BMI between the patients with 11p15 LOM and upd(7)mat.

We suggest including head and chest circumference measurements and the new chest-to-head ratio in routine testing as parameters useful in SRS clinical diagnosis.

## Supplementary Information

Below is the link to the electronic supplementary material.

Supplementary Material 1

## Data Availability

The datasets used and/or analysed during the current study are available from the corresponding author on reasonable request.

## Abbreviations

11p15 LOM: Loss of methylation *H19/IGF2*:IG-DMR at chromosome 11p15.5
95% CI: 95% confidence interval
AUC: Area under the receiver operating characteristic curve
BMI: Body mass index
CHC: Chest circumference
CHC-HC: Chest-to-head ratio
GH: Growth hormone
GP: General population
HC: Head circumference
MS-MLPA: Multiplex ligation-dependent probe amplification
NH-CSS: Netchine-Harbison clinical scoring system
ns: Not statistically significant
Q1: First quartile
Q3: Third quartile
RMC: Relative macrocephaly
ROC: Receiver operating characteristic
SDS: Standard deviation score
SGA: Small for gestational age
SRS: Silver-Russell syndrome
rhGH: Recombinant human growth hormone
upd(7)mat: Uniparental disomy of chromosome 7
