# Supplementary material for: Longitudinal study of somatic development in Polish patients with Silver-Russell syndrome reveals that chest-to-head proportion as a new clinical indicator for the syndrome
Source: Orphanet J Rare Dis. 2026 May 20;21:250. doi: 10.1186/s13023-026-04395-2 (PMC13390427; doi:10.1186/s13023-026-04395-2)
Supplement: Supplementary file 1 — Supplementary Material 1 [file 13023_2026_4395_MOESM1_ESM.docx]

Table 1. Supplement. Height, weight, weight-for-height and BMI SDS: a comparison between 11p15LOM and upd(7)mat patients in particular age categories.

| **Feature SDS** | **Wiek (lata)** | **0.25** | **1** | **2** | **3** | **4** | **5** | **6** | **7** | **8** | **9** | **10** | **11** | **12** | **13** | **14** | **15** | **17** | **Total** |
| --- | --- | --- | --- | --- | --- | --- | --- | --- | --- | --- | --- | --- | --- | --- | --- | --- | --- | --- | --- |
| Length/height  11p15  LOM | n | 49 | 135 | 103 | 81 | 85 | 73 | 70 | 59 | 43 | 28 | 29 | 23 | 20 | 22 | 11 | 13 | 32 | 876 |
|  | Me | -4.04 | -3.86 | -3.29 | -3.03 | -3.16 | -2.93 | -2.41 | -2.46 | -2.63 | -2.49 | -2.17 | -2.01 | -2.20 | -2.50 | -2.09 | -3.45 | -3.74 | - |
|  | Q1 | -4.62 | -4.83 | -3.97 | -3.78 | -3.56 | -3.74 | -3.13 | -3.01 | -3.47 | -2.95 | -2.97 | -2.50 | -2.73 | -3.38 | -2.78 | -3.94 | -4.69 | - |
|  | Q3 | -3.13 | -3.09 | -2.72 | -2.53 | -2.46 | -2.37 | -1.93 | -1.93 | -1.98 | -1.89 | -1.80 | -1.37 | -1.58 | -1.34 | -1.46 | -2.12 | -2.41 | - |
| Length/height  upd(7)mat | n | 5 | 24 | 19 | 30 | 23 | 18 | 10 | 13 | 7 | 8 | 5 | 9 | 6 | 8 | 8 | 4 | 4 | 201 |
|  | Me | -3.66 | -4.03 | -4.29 | -4.42 | -4.01 | -3.82 | -3.57 | -3.46 | -3.67 | -3.21 | -3.74 | -2.56 | -2.99 | -2.07 | -1.72 | -2.36 | -3.09 | - |
|  | Q1 | -3.67 | -5.07 | -5.75 | -4.92 | -4.61 | -4.43 | -3.81 | -3.81 | -4.28 | -3.68 | -3.77 | -3.17 | -3.13 | -3.23 | -3.63 | -3.13 | -3.60 | - |
|  | Q2 | -3.66 | -3.26 | -3.04 | -3.69 | -3.46 | -3.19 | -3.20 | -3.15 | -2.93 | -2.97 | -2.85 | -1.97 | -1.12 | -1.25 | -1.53 | -2.12 | -2.82 | - |
| - | **p** | 0.60 | 0.42 | **<0.001** | **<0.001** | **<0.001** | **0.006** | **<0.001** | **<0.001** | **0.027** | **0.003** | **0.02** | 0.11 | 0.79 | 0.73 | 0.90 | 0.48 | 0.58 | - |
| Weight  11p15  LOM | n | 107 | 179 | 129 | 89 | 87 | 76 | 71 | 60 | 45 | 28 | 30 | 23 | 21 | 22 | 11 | 13 | 32 | 1023 |
|  | Me | -3.73 | -3.39 | -3.09 | -3.14 | -2.78 | -2.52 | -2.10 | -1.71 | -1.55 | -1.78 | -1.51 | -1.76 | -2.07 | -1.69 | -1.86 | -2.52 | -2.65 | - |
|  | Q1 | -4.45 | -4.04 | -3.58 | -3.66 | -3.32 | -3.16 | -2.72 | -2.63 | -2.00 | -2.20 | -2.14 | -2.16 | -2.29 | -2.69 | -2.32 | -3.38 | -4.14 | - |
|  | Q3 | -3.07 | -2.87 | -2.67 | -2.55 | -2.25 | -2.10 | -1.78 | -1.09 | -1.18 | -1.12 | -1.02 | -0.65 | -1.63 | -1.21 | -1.63 | -2.04 | -0.53 | - |
| Weight  upd(7)mat | n | 16 | 33 | 20 | 31 | 23 | 18 | 10 | 13 | 7 | 8 | 5 | 9 | 6 | 8 | 8 | 4 | 4 | 223 |
|  | Me | -4.26 | -4.16 | -3.72 | -3.93 | -3.31 | -3.01 | -2.79 | -2.47 | -2.05 | -1.80 | -2.45 | -1.70 | -1.98 | -1.64 | -1.78 | -2.28 | -2.23 | - |
|  | Q1 | -4.65 | -4.68 | -4.46 | -4.13 | -3.48 | -3.20 | -3.00 | -2.53 | -2.34 | -1.95 | -2.46 | -2.20 | -2.31 | -2.05 | -2.38 | -2.44 | -3.26 | - |
|  | Q3 | -3.46 | -3.75 | -2.34 | -3.32 | -2.98 | -2.65 | -2.46 | -2.37 | -1.75 | -1.68 | -1.82 | -1.30 | -1.10 | -0.89 | -0.86 | -1.65 | -1.20 | - |
| - | **p** | 0.17 | **<0.001** | **<0.001** | **<0.001** | **<0.001** | 0.09 | **0.03** | **0.01** | 0.08 | 0.61 | 0.09 | 0.36 | 0.93 | 0.42 | 0.55 | 0.30 | 0.72 | - |
| Weight for height  11p15  LOM | n | - | 134 | 103 | 80 | 84 | 73 | 70 | 59 | 43 | 28 | 29 | 23 | 20 | 22 | 11 | 13 | 32 | 824* |
|  | Me | - | -1.48 | -1.96 | -1.97 | -1.77 | -1.68 | -1.40 | -0.72 | -0.79 | -1.00 | -0.30 | -0.90 | -1.19 | -1.18 | -1.22 | -1.18 | -0.75 | - |
|  | Q1 | - | -1.97 | -2.53 | -2.44 | -2.32 | -2.28 | -1.99 | -1.67 | -1.54 | -1.59 | -1.22 | -1.33 | -1.53 | -1.40 | -1.54 | -1.59 | -1.49 | - |
|  | Q3 | - | -1.16 | -1.54 | -1.54 | -1.08 | -1.20 | -0.58 | 0.06 | 0.00 | -0.02 | 0.19 | 0.49 | -0.13 | -0.13 | -0.61 | -1.01 | 1.02 | - |
| Weight for height  upd(7)mat | n | - | 24 | 19 | 30 | 23 | 18 | 10 | 13 | 6 | 9 | 5 | 9 | 6 | 8 | 8 | 4 | 4 | 196* |
|  | Me | - | -2.13 | -2.37 | -2.15 | -1.89 | -1.70 | -1.65 | -1.35 | -1.12 | -0.78 | -1.29 | -0.61 | -0.71 | -0.34 | -0.22 | -0.58 | -0.06 | - |
|  | Q1 | - | -2.55 | -2.63 | -2.32 | -2.17 | -2.26 | -2.14 | -1.83 | -1.23 | -1.07 | -1.29 | -0.79 | -0.92 | -0.56 | -0.79 | -1.21 | -0.79 | - |
|  | Q3 | - | -1.79 | -2.10 | -1.83 | -1.62 | -1.53 | -1.15 | -1.18 | -0.90 | -0.32 | -0.93 | -0.28 | -0.48 | -0.18 | -0.14 | 0.07 | 0.59 | - |
| - | **p** | **-** | **0.001** | **0.02** | 0.58 | 0.30 | 0.59 | 0.33 | 0.07 | 0.40 | 0.83 | 0.21 | 1.00 | 0.56 | 0.23 | **0.04** | 0.28 | 0.41 | - |
| BMI | n | 49 | 133 | 102 | 80 | 83 | 73 | 69 | 54 | 42 | 27 | 27 | 21 | 19 | 20 | 11 | 12 | 28 | 850 |
| 11p15 | Me | -2.19 | -2.22 | -2.41 | -2.27 | -1.79 | -1.86 | -1.73 | -1.42 | -0.98 | -1.40 | -0.96 | -1.70 | -1.45 | -1.81 | -1.82 | -2.20 | -2.45 | - |
| LOM | Q1 | -2.72 | -2.73 | -3.09 | -3.05 | -2.41 | -2.35 | -2.49 | -2.24 | -1.51 | -1.95 | -2.00 | -2.09 | -2.17 | -2.55 | -2.25 | -3.13 | -3.37 | - |
|  | Q3 | -1.70 | -1.52 | -1.87 | -1.58 | -1.10 | -1.38 | -0.89 | -0.34 | -0.26 | -0.19 | 0.02 | 0.06 | -0.67 | -0.77 | -1.14 | -1.83 | -0.52 | - |
| BMI | n | 4 | 24 | 19 | 30 | 23 | 18 | 10 | 13 | 7 | 8 | 5 | 9 | 6 | 8 | 8 | 4 | 4 | 200 |
| upd(7)mat | Me | -2.18 | -2.97 | -2.77 | -2.61 | -1.93 | -1.75 | -1.70 | -1.67 | -1.19 | -1.16 | -1.66 | -1.21 | -1.23 | -0.95 | -1.17 | -1.43 | -0.84 | - |
|  | Q1 | -2.47 | -3.50 | -4.05 | -2.81 | -2.26 | -2.23 | -2.43 | -1.88 | -1.64 | -1.36 | -1.76 | -1.45 | -1.75 | -1.30 | -1.54 | -2.05 | -2.13 | - |
|  | Q2 | -1.76 | -2.63 | -1.16 | -1.82 | -1.42 | -1.48 | -1.07 | -1.33 | -1.04 | -0.61 | -1.18 | -0.70 | -0.82 | -0.49 | -0.39 | -0.58 | 0.26 | - |
| - | **p** | 0.83 | **<0.001** | 0.27 | 0.75 | 0.52 | 0.75 | 0.91 | 0.41 | 0.42 | 0.52 | 0.34 | 0.59 | 0.56 | 0.07 | 0.05 | 0.17 | 0.31 | - |

n-number of measurements, *weight for height – without 0.25 y., because of number of measurements (11p15LOM – 25, upd(7)mat – 2)
